# Victims of eugenic sterilisation in Utah: cohort demographics and estimate of living survivors

**DOI:** 10.1016/j.lana.2023.100436

**Published:** 2023-02-15

**Authors:** James Tabery, Nicole L. Novak, Lida Sarafraz, Aubrey Mansfield

**Affiliations:** a Department of Philosophy, University of Utah, USA; b Department of Community and Behavioral Health and Public Policy Center, University of Iowa, USA; c Department of Philosophy, University of Utah, USA; d College of Health, University of Utah, USA

**Keywords:** Eugenics, Sterilisation, Reproductive justice, Medical ethics, Disability, Health law, Genetics

## Abstract

**Background:**

Eugenicists at the beginning of the twentieth century feared that the “unfit” were outbreeding the “fit” and promoted interventions like sterilisation as a solution to the perceived problem. Over 60,000 people were sterilised across the United States, victims of eugenic programs implemented in 32 states. Utah had a particularly aggressive eugenic sterilisation program, hailed by eugenicists for sterilising such a large proportion of its population, and lasting well into the 1970s. The goal of the present study was to determine who, at the demographic level, was targeted by this eugenic practice in Utah, and to also estimate how many survivors of the program might still be alive in 2023.

**Methods:**

We used archival records and data abstracted from charts at the Utah State Developmental Center to construct an observational cohort of people sterilised under Utah's coercive, eugenic sterilisation program. We described the demographics of the cohort and presented a life table analysis to estimate the number of survivors still living in 2023.

**Findings:**

At least 830 men, women, and children (modal age of 15–19, 53.6% female) were sterilised in Utah institutions under a program that was launched in 1925, peaked in the 1940s, and concluded in the 1970s. The life table analysis predicts approximately 54 survivors (36 women, 18 men), with an average age of 78.

**Interpretation:**

Many people sterilised under Utah's eugenics law are likely living today. While some states have taken steps to reckon with their roles in depriving people of their reproductive rights, Utah lacks even an official acknowledgment of this shameful, medical history. Given the advanced age of the potential survivors, time is running out for a reconciliation that can be experienced by those who were most harmed by the practice.

**Funding:**

This research was supported by three grants from the 10.13039/100000051National Human Genome Research Institute at the U.S. 10.13039/100000002National Institutes of Health (RM1HG009037, R25HG010020, R01HG010567).

**Research in context**

**Evidence before this study**

The abhorrent history of eugenics is well known to the medical and public health communities. Scholars have revealed the ableist, racist, nativist, and sexist ideas that undergirded eugenic fears about the “unfit” outbreeding the “fit.” They have documented the crude notions of heredity utilized to justify efforts at eradicating criminality, poverty, and intellectual disability. And they have revealed how those unscientific biases infiltrated the legal, political, and medical realms, leading to laws permitting coercive sterilisation, prohibitions on who could immigrate into a country, and paternalistic proscriptions about who should not marry or reproduce. Though well known, this history is also often mistakenly assumed to be a relic of the distant past—a product of the early-twentieth century that ceased to exist, both in terms of ideology and harms done to individuals, at the end of World War II. Researchers have devoted increasing attention to state- and institution-level analyses of this history, allowing for a closer examination of the people harmed by the eugenic assault on their reproductive autonomy and permitting a better understanding of who was targeted, how that changed over time, and how long it lasted.

**Added value of this study**

This study provides information about the human scale of eugenic sterilisation in one state in the U.S.—Utah—where eugenic ideas and practices continued long after the 1940s. Sterilisations were sanctioned at four state institutions and continued at one institution well into the 1970s. That was reason to think that there could still be survivors alive in 2023. We provide sociodemographic information about nearly every victim of a sterilisation program that operated across six decades, many of whom were children when they were sterilised (one who was under ten years old), and also estimate there to be approximately 54 survivors alive today.

**Implications of all the available evidence**

An “anti-eugenic future” can only be achieved, authors of an article in *The Lancet* pointed out, if the health community specifically and the public more generally honestly confronts its eugenic past. Attempts to reckon with that painful history have taken various forms: official apologies, truth commissions, historical markers, restitution programs. Many of those efforts began by first acknowledging the human scope of those programs—both the victims who are gone, and the survivors who still live with the scars of the eugenic assault on their reproductive liberties.

## Introduction

When a teenage girl, in 1928, told her local religious leader that she'd been repeatedly raped by a family member, the man did not believe her. Instead, she was admitted to the Utah State Hospital, diagnosed as a “moron,” and sterilised. After her release, that same religious leader admitted she was probably being sold as a sex worker by another family member.^1^ Forty-five years later, a teenage boy at the Utah State Training School learned that he was scheduled for sterilisation. His initial reaction was violent objection, motivated by his desire to have children. As time went on, however, he'd resigned himself to the fact that there was little he could do to prevent the operation.^2^ These two individuals were among the first and last victims of state-sanctioned, eugenic sterilisation in Utah.

Eugenicists at the beginning of the twentieth century perceived a grave threat to the nation; the “unfit,” they warned, were outbreeding the “fit.”^3^^,^^4^ Eugenics combined a naïve view of human genetics with discriminatory ideas about who should and shouldn't reproduce.^5^ People with psychiatric diagnoses, physical and intellectual disabilities, as well as those who were incarcerated or who were deemed to be sexually deviant were all targeted by eugenicists as economic and moral threats to society, the solution to which were sterilisation programs intended to prevent them from transmitting their traits to progeny.^6^ Indiana, in 1907, was the first U.S. state to enact legislation permitting eugenic sterilisations, and the practice spread widely after the 1927 ruling of the U.S. Supreme Court in *Buck v. Bell*, which deemed such surgeries to be constitutional.^7^ All told, 32 states passed laws that allowed for the sterilisation of over 60,000 people living in the United States.^8^

Scholars of this dark chapter in medicine have devoted increasing attention to state- and institution-level analyses of this history because it allows for examining more closely the people harmed by this assault on reproductive autonomy: who was targeted, how that changed over time, how long it lasted.^8^, ^9^, ^10^, ^11^, ^12^, ^13^, ^14^, ^15^, ^16^, ^17^, ^18^, ^19^ Utah presents a particularly striking case for such an investigation because, though the total number of people sterilised in the sparsely populated state was smaller than states like California and North Carolina, it had an extremely aggressive sterilisation program. Eugenicists, in fact, hailed Utah for sterilising a far greater proportion of its residents than any other state in 1947 as an “important achievement in public health.”^20^ The Utah state legislature passed its sterilisation bill in 1925, allowing for the sterilisation of anyone institutionalised at the Utah State Hospital, Utah State Prison, or Utah State Industrial School and deemed to be “habitually sexually criminal, insane, idiotic, imbecile, feeble-minded, or epileptic and by the laws of heredity is the probable parent of socially inadequate off-spring likewise afflicted.”^21^ After the Utah State Training School opened in 1931 for the explicit purpose of caring for those judged to be “feebleminded,” the law was revised to include patients there too.^22^ Utah's program included a notification period, hearing, “consent” process, and appeal mechanism; however, it is clear from interviews conducted with patients that this process would not meet contemporary standards for informed consent. Consent forms were not required to be from the patient themself, and many who were targeted did not want to undergo the procedure or did not even understand that they had been or were going to be sterilised.^23^ In some cases, requirement of sterilisation for discharge from an institution added further coercive pressure.^24^

Utah is also rather striking because sterilisations continued to be performed there for fifty years. Throughout the 1940s and 1950s it became increasingly clear that human genetics didn't provide a scientific rationale for eliminating disabilities or criminality by sterilising people.^25^ The Utah legislature, however, worked around that development by changing the rationale for its sterilisation program in 1961.^26^ The original formulation of the law required that people the state deemed “unfit” could be sterilised if they were judged likely to pass what were thought to be hereditary traits on to their offspring, but the evaporating genetic justification for that rationale restricted the extent to which the sterilisation program could function.^27^^,^^28^ So the lawmakers swapped out the genetic justification with a new, less restrictive rationale for sterilisation, arguing that people could be sterilised if they were determined “unlikely to be able to perform properly the functions of parenthood… and that the welfare of the inmate or person and of society will be promoted by such sterilization or asexualization.” As one newspaper from the era summarised the legislative shift, “Instead of having to prove a genetic defect, it is now necessary only to show that a person is not and has no chance of becoming a fit parent.”^29^ With this new justification codified into law, coercive sterilisations continued to be performed well into the 1970s. This fact suggested that Utah's program may have ceased decades ago, but the historical injustice associated with it could live on in the bodies of sterilisation survivors still alive today. The purpose of this study was to determine who, at the sociodemographic level, was subjected to this reproductive oppression and to estimate how many survivors might still be alive in 2023.

## Methods

### Sterilisation records

This analysis draws on two sources of sterilisation data to generate an observational cohort of people sterilised under Utah's state eugenics law. The primary source is a deidentified database compiled from a chart review at the Utah State Developmental Center (USDC; formerly the Utah State Training School). Through a formal agreement with the University of Utah, USDC staff systematically abstracted sociodemographic information from all available sterilisation records, which spanned the years 1932–1974 (n = 849).^30^ Recorded variables included year of birth, sex, race/ethnicity, marital status, occupation, ascribed IQ, and years of admission, sterilisation, discharge, and death. Due to USDC data privacy restrictions, year of birth was not provided for patients born prior to 1929, and sterilisation years were presented in 3-year ranges as opposed to precise sterilisation year. USDC staff also reviewed USDC records, public obituaries, and Utah cemetery records to seek evidence of a year of death. Data entry took place from 2019 to 2021. The second data source is a 1932 master's thesis from the University of Utah by Gordon Sears, which includes narrative documentation of 77 sterilisations performed in Utah prior to 1932.^1^ A trained research assistant reviewed each case in the Sears database and abstracted available, deidentified information on the same variables as the USDC files. The collection and use of the USDC data in the study design was approved by the Institutional Review Boards of the University of Utah (IRB_00121,214) and the State of Utah Department of Human Services (0660). The use of the deidentified, publicly available data in the Sears thesis qualified as non-human subjects research by the University of Utah (IRB_00159,898).

Variables included in the present analysis are year of birth, sex (male, female), race/ethnicity (recorded as listed in the file and later classified as non-Hispanic White, non-Hispanic Black, Hispanic/Latino, Asian, Multiracial), admission year, sterilisation year, discharge year and year of death. In the present analysis, sterilisation years were imputed as the middle year in the 3-year range provided.

**Fig. 1:**
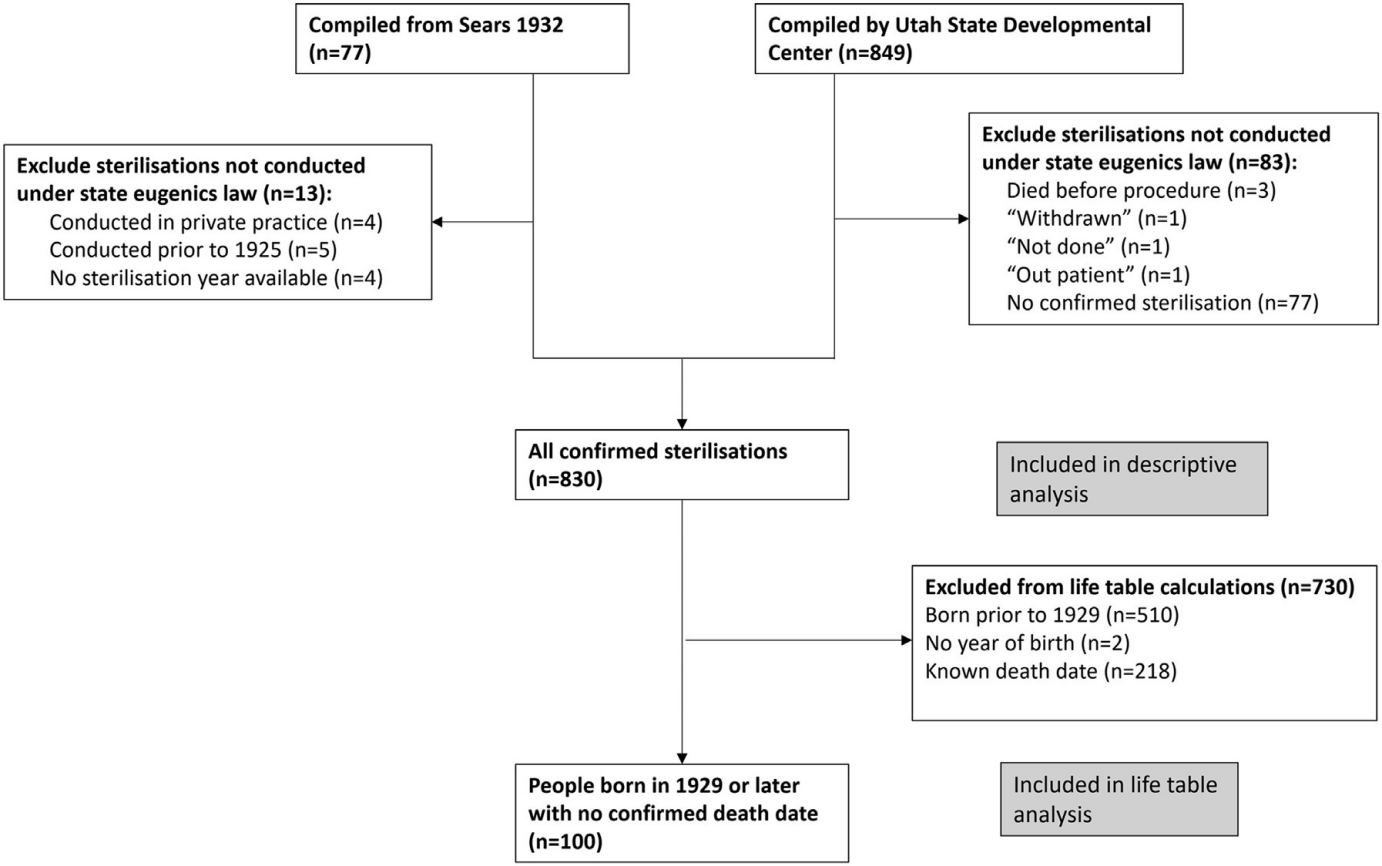
Flow diagram depicting all sterilisation records available from the two data sources, number of records excluded from descriptive analysis, and number of records excluded from the life table analysis.

Fig. 1 presents a flow diagram of data included in the present analysis. Of 849 records in the USDC database, 83 were excluded either because there was not confirmed documentation of the sterilisation taking place (n = 77), or because the procedure was never performed (n = 6), leaving 766 confirmed sterilisations from the USDC database. Of 77 records in the Sears database, 13 were excluded either because they were conducted prior to the state eugenics law's passage in 1925 (n = 5), there was no confirmed year of sterilisation (n = 4), or they were conducted in private practice (n = 4), leaving 64 confirmed sterilisations from this data source. Combining these two data sources, there were 830 confirmed sterilisations performed under Utah's state eugenics law and included in the descriptive analysis.

Additional exclusions were applied to the data prior to life table analysis, which requires information on year of birth. The USDC database had redacted year of birth prior to 1929, so all persons born prior to 1929 were excluded (n = 510). Also excluded from the life table analysis were persons with no known year of birth (n = 2) and all persons with a known death date (n = 218), leaving 100 people born in 1929 or later with no confirmed death date.

The life table analysis used three variables: sex, year of birth, and most recent date confirmed alive. For 96 people, this was the discharge date from the institution. For 4 people with no confirmed discharge date, year of sterilisation was used as the most recent date alive.

### Analytic methods

Descriptive analysis of all confirmed sterilisations (n = 830) included frequencies and descriptive statistics for sociodemographic characteristics, stratified by data source.

### Demographic methods

Sex-specific life tables were used to estimate the annual survival probability of people confirmed to be sterilised under Utah's state eugenics law. This analysis used the National Center for Health Statistics (NCHS) decennial (10-year) life tables for Utah from 1940 to 2000, and the decennial life tables for the total United States population for 2010. For 1940–1960, life tables were only available by specific racial category; life tables for the white population were used. Life tables for the general population (regardless of race) were used from 1970 to 2010. Age-specific death probabilities reported in decennial life tables were extended 5 years before and after the date of the decennial table. For example, death probabilities reported in the 1950 life table were applied to years 1946–1955. Death probabilities from the decennial life table for the United States in 2010 were applied from 2005 to 2023, as state-specific decennial life tables for 2010 and 2020 have not yet been released.^31^, ^32^, ^33^

Age-, sex- and decennial-specific death probabilities were subtracted from one to generate the probability of each individual surviving from their age at sterilisation (age *a)* to their age one year later (*a* + 1).^34^ This was then multiplied by the probability of them surviving from that year (age *a* + 1) to the following year (*a* + 2), using probabilities from the life table that corresponded to age *a* + *1*. This process continued, adding in new individuals to the cohort of potential survivors, for every year to 2023.

### Role of the funding source

This research was supported by three grants from the 10.13039/100000051National Human Genome Research Institute at the 10.13039/100000002U.S. National Institutes of Health (RM1HG009037, R25HG010020, R01HG010567). The funders of this study played no role in study design, data collection, data analysis, interpretation, or writing of the report.

## Results

**Fig. 2:**
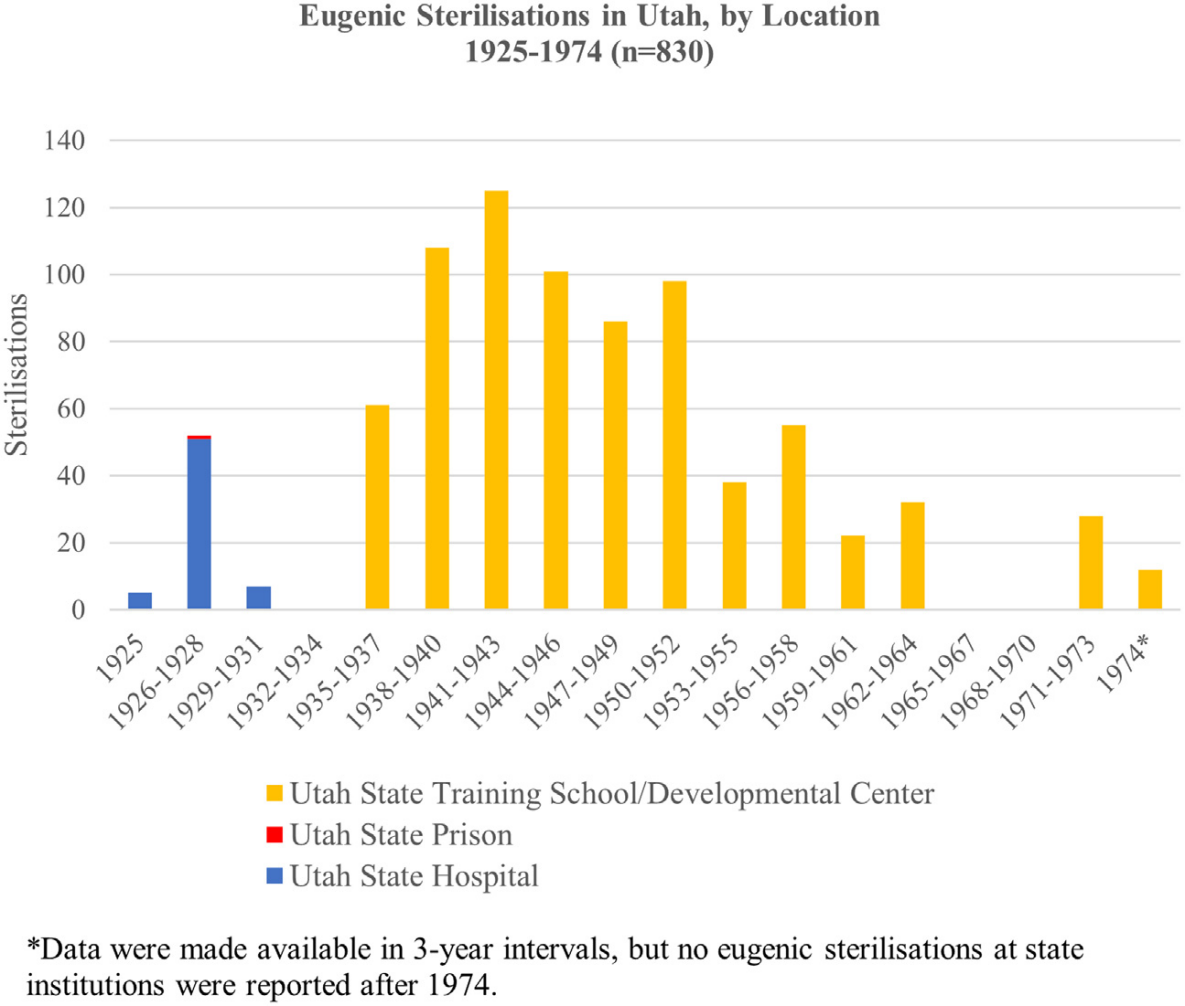
Timeline of Utah's sterilisations recorded in archival records, 1925–1974.

Fig. 2 depicts triannual counts of sterilisations performed under Utah's state eugenics law, according to the location where the sterilisation was performed. The majority took place at the Utah State Training School/Developmental Center from 1932 to 1974, followed by the Utah State Hospital from 1925 to 1931. One sterilisation was performed at the Utah State Prison.^35^ Annual sterilisations peaked between the late 1930s through the early 1950s, with 125 sterilisations performed between 1941 and 1943.

Table 1 describes the 830 people sterilised under Utah's state eugenics law, by data source and overall. The modal age category was 15–19 (23.9% of cases). Slightly more than half of cases (53.6%) were female. Non-Hispanic White people were the largest racial/ethnic group (80.1%, with an additional 18.1% missing information on race/ethnicity).

**Table 1:** Descriptive statistics, sterilisations performed under Utah state eugenic sterilisation law, 1925–1974 (n = 830).

	Data source		
	Sears 1932 (n = 64)	Utah state developmental center (n = 766)	Total (n = 830)
	N (%)	N (%)	N (%)
Approximate age at sterilisation (calculated)			
<10	–	1 (0.1%)	1 (0.1%)
10–14	–	50 (6.5%)	50 (6.0%)
15–19	11 (17.2%)	187 (24.4%)	198 (23.9%)
20–29	29 (45.3%)	80 (10.4%)	109 (13.1%)
30–39	17 (26.6%)	–	17 (2.1%)
40–49	6 (9.4%)	–	6 (0.7%)
No year of birth	1 (1.6%)	448 (58.5%)	449 (54.1%)
Sex			
Male	35 (54.7%)	349 (45.6%)	384 (46.3%)
Female	29 (45.3%)	416 (54.3%)	445 (53.6%)
Missing	–	1 (0.1%)	1 (0.1%)
Race/ethnicity			
Non-Hispanic White	–	665 (86.8%)	665 (80.1%)
Non-Hispanic Black	–	2 (0.3%)	2 (0.2%)
Hispanic/Latino^a^	–	10 (1.3%)	10 (1.2%)
Asian	–	1 (0.1%)	1 (0.1%)
Multiracial^b^	–	2 (0.3%)	2 (0.2%)
Missing	64 (100%)	86 (11.2%)	150 (18.1%)

a Among people classified as Hispanic/Latino: 3 Mexican-origin, 1 Puerto Rican, 1 “white/Hispanic”, 1 “White and Spanish”.

b Among people classified as multiracial: 1 “White/Pacific Islander”, 1 “White/Native American”.

The life table analysis included 61 women and 39 men. Applying standard sex-specific survival probabilities through to the present, there would be an estimated 36 women and 18 men surviving in 2023, for a total of 54 living survivors. The average age for survivors in 2023 is estimated to be 78. Relative to the total population originally recommended for sterilisation, the estimated survivor group has a higher proportion of females, a younger average age at sterilisation, and a later date of sterilisation.

## Discussion

The comprehensive nature of the data used for these analyses is noteworthy, particularly because there was no single eugenics board overseeing all sterilisations in Utah for the duration of the practice. Coercive, eugenic sterilisations took place in Utah across six decades and in three different institutions. (No sterilisations were reported at the Utah State Industrial School even though it was authorised to perform them.) Of all the documented cases of eugenic sterilisation across that time and space, only about two dozen are missing from the present analysis, providing a nearly complete profile of the patients who were targeted in Utah.^36^ It is also worth highlighting that this data allowed for analysing data on people who were confirmed to have been sterilised, rather than data from people who were proposed for sterilisation but who may or may not have been ultimately sterilised. Information on date of discharge and date of death also made the records included in this analysis more comprehensive than those available in other states.^16^

Although the data used in this analysis is unique and robust, there are several limitations to the life table analysis used to estimate the number of living survivors. Using survival probabilities from the general population of Utah likely overestimates the number of actual survivors, since the individuals who were sterilised were disproportionately likely to experience congenital or chronic health issues, poverty, and other risk factors that are associated with a shorter life expectancy than the general population.^37^^,^^38^ On the other hand, excluding from analysis the people with no confirmed sterilisation date may underestimate the number of actual survivors, as the absence of a sterilisation date does not guarantee that a person wasn't sterilised, only that their sterilisation could not be confirmed. Furthermore, excluding all observations with known death dates could have restricted the analysis to a group of survivors who are disproportionately healthy (survivor bias). Although there is uncertainty associated with the estimated number of living survivors, existing methods to generate confidence intervals for life table estimates apply very small standard errors generated from population mortality data. The bidirectional sources of potential error described above are not as readily quantified: we do not know the distribution of risk factors for poor health in the study population, nor do we know the magnitude of any potential survivor bias in the subset of survivors with no confirmed death data. However, the estimated 54 living survivors gives a sense for the order of magnitude of potential living survivors today, making clear that the history of eugenics is not long past, and that many survivors of Utah's eugenic sterilisation program are likely living today.

The medical and public health community has grown increasingly attuned to the legacies of historical injustices in health and medicine. The COVID-19 pandemic has underscored the enduring marginalisation of people with disabilities in public health practice.^39^ Histories of coerced sterilisation of people with disabilities add moral urgency to current efforts for reproductive freedom among people with disabilities, who continue to experience higher rates of sterilisation than nondisabled people and who continue to be subjected to unconsented sterilisation in many U.S. states.^40^^,^^41^ Shining a bright light on these painful histories allows for assessing the toll taken on marginalised communities, with an eye towards rectifying past harms and preventing their perpetuation.^42^ The commonly held myth that eugenic ideas and eugenic practices ended at the conclusion of World War II is undermined by the assault on reproductive liberties that took place in the form of coercive sterilisations at state and federal institutions for decades after.^43^ An interdisciplinary and international team of scholars pointed out that an “anti-eugenic future” can only be achieved when the medical community and the wider public confronts this eugenic past and reckons with what is owed to survivors, a powerful reminder to be “vigilant for the danger of repetition.”^44^

A number of states in the U.S. have taken steps to begin reckoning with their government's official role in depriving their people of reproductive rights. Several governors, in 2002 and 2003, apologised to the victims of eugenic sterilisation in their states.^45^^,^^46^ The Vermont legislature expressed similar remorse in 2021.^47^ North Carolina, Virginia, and California have gone even further with compensation programs.^48^ Utah, on the other hand, has expressed no such regret nor offered any such restitution to its population of sterilisation victims, lacking even an official acknowledgement of this shameful history.

The case for such reconciliation efforts is strong. Eugenic sterilisation programs were scientifically unfounded and morally reprehensible; they did real harm to members of already-marginalised communities, permanently stealing their right to decide for themselves whether to have children and deeply shaming them by singling them out as unworthy of that right. Public apologies from state officials, even if those who express remorse were not involved in the original harm, can serve to redirect shame away from the victims and toward the government forces that took advantage of their vulnerable status; they can make plain for both victims and the wider public that a grave mistake was made and must be guarded against so that it is not repeated, and they can create space for survivors to come forward and reveal the true human costs of such programs.^45^^,^^47^ Compensation programs for survivors of eugenic sterilisation are justified as restitution for the injury perpetuated by the state.^49^ Precedent can be found in similar cases where compensation programs were created in response to specific government abuses that targeted specific populations, such as victims of the U.S. government's Japanese internment camp system.^50^

The stubborn persistence of Utah's sterilisation program was particularly egregious. When it became abundantly clear that genetic principles did not support the practice, a natural response would have been to terminate the program as other states did. Instead, legislators concocted a new rationale in 1961 designed to allow the program to continue sterilising people with what was thought to be a lower scientific threshold—that those who were unfit to parent could be distinguished from those who were fit.^27^, ^28^, ^29^ Given the advanced age of potential survivors in Utah, many of whom were likely sterilised after the 1961 update to Utah's sterilisation legislation, time is running out for a reconciliation that can be experienced by those who were most harmed by the medical practice.

## Contributors

James Tabery contributed the following: conceptualisation, investigation, funding acquisition, writing—original draft, review and editing, supervision, and project administration. Nicole Novak contributed the following: conceptualisation, investigation, methodology, formal analysis, data curation, funding acquisition, writing—original draft, review and editing, visualisation, and validation. Lida Sarafraz contributed the following: data curation, resources, and writing—review and editing. Aubrey Mansfield contributed the following: resources and writing—reviewing and writing.

## Data sharing statement

Dataset is available from the authors upon request.

## Declaration of interests

Authors declare no competing interests.
